# Establishment and validation of a prediction model for self-absorption probability of chronic subdural hematoma

**DOI:** 10.3389/fneur.2022.913495

**Published:** 2022-07-22

**Authors:** Ye Tian, Dong Wang, Xinjie Zhang, Huijie Wei, Yingsheng Wei, Shuo An, Chuang Gao, Jinhao Huang, Jian Sun, Rongcai Jiang, Jianning Zhang

**Affiliations:** ^1^Department of Neurosurgery, Tianjin Medical University General Hospital, Tianjin, China; ^2^Key Laboratory of Post-Neurotrauma Neurorepair and Regeneration in Central Nervous System, Ministry of Education in China and Tianjin, Tianjin, China; ^3^Tianjin Key Laboratory of Injury and Regenerative Medicine of Nervous System, Tianjin, China; ^4^Department of Neurosurgery, Tianjin Neurological Institute, Tianjin, China

**Keywords:** chronic subdural hematoma, self-absorption, non-surgical treatment, prediction model, nomogram

## Abstract

**Background:**

Chronic subdural hematoma (CSDH) is common in elderly people with a clear or occult traumatic brain injury history. Surgery is a traditional method to remove the hematomas, but it carries a significant risk of recurrence and poor outcomes. Non-surgical treatment has been recently considered effective and safe for some patients with CSDH. However, it is a challenge to speculate which part of patients could obtain benefits from non-surgical treatment.

**Objective:**

To establish and validate a new prediction model of self-absorption probability with chronic subdural hematoma.

**Method:**

The prediction model was established based on the data from a randomized clinical trial, which enrolled 196 patients with CSDH from February 2014 to November 2015. The following subjects were extracted: demographic characteristics, medical history, hematoma characters in imaging at admission, and clinical assessments. The outcome was self-absorption at the 8th week after admission. A least absolute shrinkage and selection operator (LASSO) regression model was implemented for data dimensionality reduction and feature selection. Multivariable logistic regression was adopted to establish the model, while the experimental results were presented by nomogram. Discrimination, calibration, and clinical usefulness were used to evaluate the performance of the nomogram. A total of 60 consecutive patients were involved in the external validation, which enrolled in a proof-of-concept clinical trial from July 2014 to December 2018.

**Results:**

Diabetes mellitus history, hematoma volume at admission, presence of basal ganglia suppression, presence of septate hematoma, and usage of atorvastatin were the strongest predictors of self-absorption. The model had good discrimination [area under the curve (AUC), 0.713 (95% *CI*, 0.637–0.788)] and good calibration (*p* = 0.986). The nomogram in the validation cohort still had good discrimination [AUC, 0.709 (95% *CI*, 0.574–0.844)] and good calibration (*p* = 0.441). A decision curve analysis proved that the nomogram was clinically effective.

**Conclusions:**

This prediction model can be used to obtain self-absorption probability in patients with CSDH, assisting in guiding the choice of therapy, whether they undergo non-surgical treatment or surgery.

## Introduction

Chronic subdural hematoma (CSDH) is common in elderly people, which is combined with clear or occult traumatic brain injury (TBI) history (1). Its incidence is 127.1/100,000 among patients of 80 years or older (2). Traditional treatment has normally involved surgery to remove the hematomas (3), but the recurrence rate among high-risk patients is approximate 25.6% (4) and mortality rate among elderly is approximately 24–32% (5). Non-surgical treatment may be more effective and safer on some patients with CSDH. Recently, we accomplished a randomized controlled trial (Efficacy of Atorvastatin on Chronic Subdural Hematoma, ATOCH) (6). The results showed the hematoma in 76.5% (75/98) of patients with CSDH in the placebo group would be self-absorption, but the self-absorption time of different patients varied obviously, from several weeks to several months. Atorvastatin, an HMG-CoA reductase inhibitor, could increase the self-absorption rate to 88.8% (87/98), and shorten the average absorption time. But it is a challenge to speculate which parts of patients could obtain benefits from non-surgical treatment. Meanwhile, it would be unsafe and rewardless for the patients without self-absorption ability to use non-surgical treatment. Evaluating the self-absorption ability of different patients with CSDH could guide the choice of treatment. Based on current knowledge, it is a fact that no research specified which kind of factors would enable superior prediction of self-absorption. Therefore, the aim of this study was to establish and validate a prediction model for self-absorption probability in patients with chronic subdural hematoma. On this basis, a more evidence-based approach could contribute to clinical practice guidance.

## Materials and methods

### Source of data

All patients had provided written consents before enrollment. This study re-analyzed the entire database with the approval of the ethics committee of Tianjin Medical University General Hospital. The primary cohort of this study from a randomized controlled trial (ATOCH) (6), which was gathered from February 2014 to November 2015, was used to develop the prediction model for self-absorption probability in patients with CSDH. ATOCH was a randomized clinical trial of atorvastatin on the absorption effect of hematoma among 196 individuals with CSDH (169 men and 27 women; median age, 65 (54.5–75) years; range, 24–89 years). In total, 60 consecutive outpatient CSDHs (45 men and 15 women; mean age, 66.5 (58.5–75 years; range, 34–89 years) as the independent validated cohort were enrolled in a proof-of-concept clinical trial from July 2014 to December 2018 by the same researchers (7).

We included patients with the following characteristics: no surgery treatment, first onset of CSDH, and lack of anticoagulant treatment. The following exclusion criteria was adopted to all study samples: CSDH caused by cancer, hemopathy, or other known serious comorbidities, and lack of follow-up at the 8th week.

### Clinical data

The following clinical statistics were gathered: age, sex, history of TBI, history of hypertension, diabetes mellitus (8) and hyperlipemia, Markwalder grading scale/Glasgow Coma Scale (MGS-GCS), activities of daily living (9) at admission, and the use of atorvastatin.

### Images acquisition and analysis

**Figure 1:**
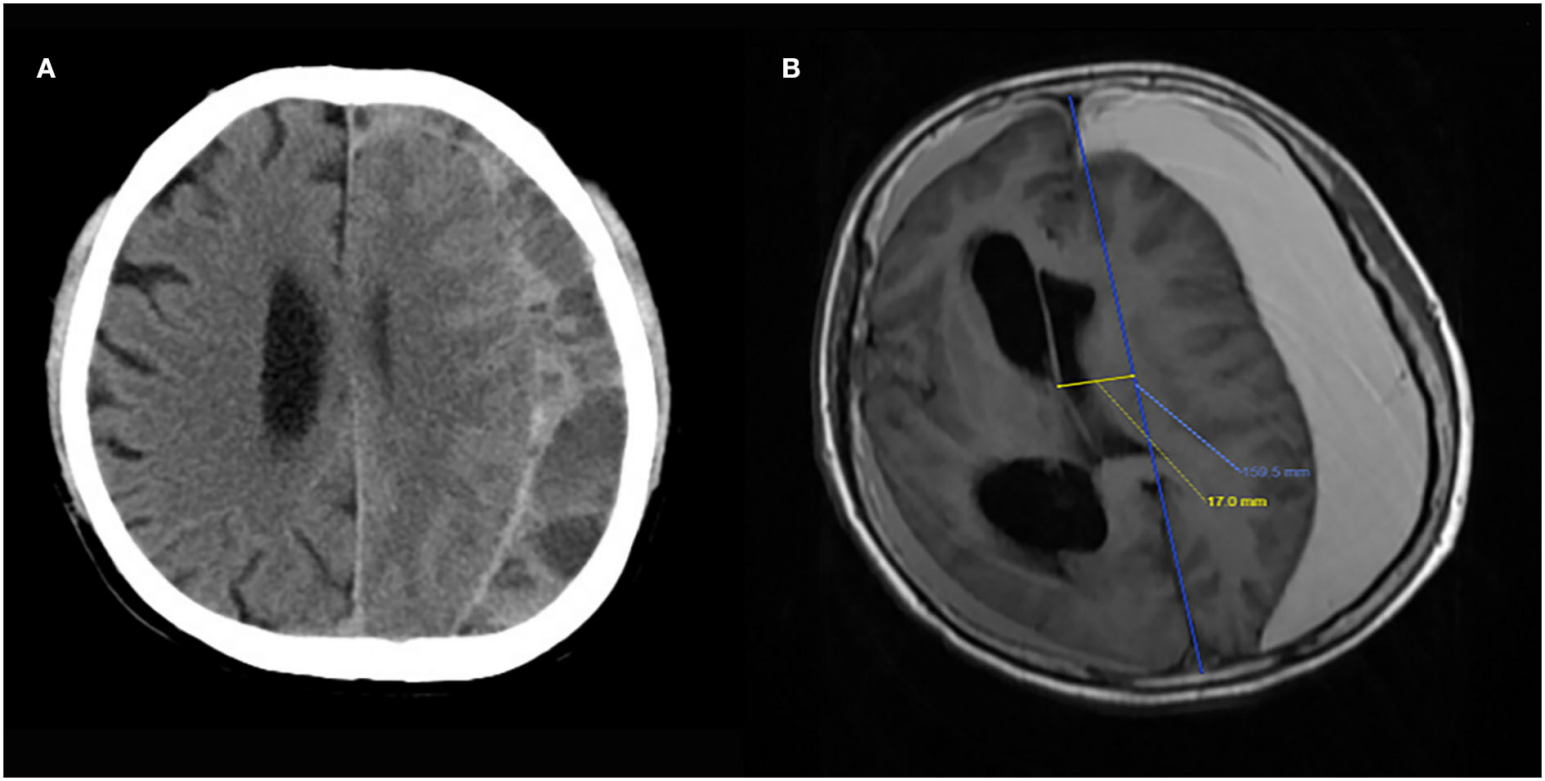
Image examples of the key features of presence of septation **(A)** and basal ganglia compression **(B)**.

CT images were collected with different scanners at each center and sent electronically to Tianjin Medical University General Hospital. All the images were uniformly analyzed by 3 independent neuroradiologists to standardize the measurements. The following imaging data were record: hematoma volume (calculated using the Tada formula), thickness of hematoma, midline shift distance, presence of basal ganglia compress, hematoma location (unilateral or bilateral hematoma), and presence of septate hematoma (Figure 1). Hematoma volume was calculated using the Tada formula and received the results after an average of three neuroradiologists' calculations. All patients underwent a follow-up CT scan at 8 weeks after enrollment.

### Outcome

Whether hematoma could be self-absorbed without surgery at the 8th week was the outcome of this prediction model. Good absorption was defined as hematoma reduction >50% from baseline hematoma volume. Poor absorption was defined as hematoma reduction <50% or hematoma enlargement.

### Statistical analysis

#### Comparison between good absorption and poor absorption group in primary and validation cohort

Following the evaluation of the normal distribution by Shapiro–Wilk testing, the data were summarized using the mean with SD or the median with quartiles as continuous variables, and the classification variables were summarized using the frequency and percentage. The comparison between good absorption and poor absorption groups in the primary and validation cohorts was analyzed by Student's unpaired *t*-test, Wilcoxon test, or chi-square test. All statistical analyses were conducted using the SPSS version (SPSS Inc., Chicago, Ill), with *p* < 0.05 given statistical meaning.

#### Feature selection and establishment of an individualized prediction model

The least absolute shrinkage and selection operator (LASSO) method (10) was used to choose the most effective predictive features from the primary dataset. The following features were selected and analyzed by multivariable logistic regression: history of diabetes mellitus (8), the use of atorvastatin (drug), hematoma volume (volume), presence of basal ganglia compress (compress), and presence of septate hematoma (separate). Subsequently, a predictive model of CSDH self-absorption was established based on the multivariable logistic analysis of the primary cohort. To provide clinicians with a quantitative tool to predict the probability of individual CSDH self-absorption, we constructed the nomogram based on the predictive model in the primary cohorts described above. We have considered the interaction between hematoma volume and basal ganglia compress in the nomogram. The LASSO method and the nomogram were conducted with R software (version 3.3.0), and multivariable logistic analysis was been analyzed with the help of SPSS (version 19).

#### Apparent performance of the nomogram in the primary cohort

Calibration curves were drawn to evaluate the alignment of the nomogram by Hosmer–Lemeshow tests (11). A receiver operating characteristic (ROC) curve was measured to quantify the discrimination performance of the nomogram (12). Nomograms and calibration curves were conducted with R software (version 3.3.0), and an ROC curve was performed using SPSS (version 19).

#### Independent validation of the nomogram

The validation cohort helped to inspect the performance of the internally validated nomogram. The predictive model formed in the primary cohort lends itself to all patients in the validation cohort and helps to digitalize the total score for every single patient. The ROC curve and calibration curve were then performed on the factors of the total score.

### Clinical use

A decision curve analysis was managed to evaluate the clinical effectiveness of the nomogram, which quantified the net benefits at different threshold probabilities in the primary cohort (13).

## Results

In Table 1, information was stated including the baseline demographic, clinical, and imaging characteristics between good absorption and poor absorption groups in the primary and validation cohorts. The good absorption rate was 66.3 and 65% in the primary and validation cohorts, respectively. No obvious difference was found in the baseline characteristics between two cohorts, either within the good absorption or in the poor absorption group.

**Table 1:** Characteristics of patients with chronic subdural hematoma (CSDH) in the primary and validation cohorts.

**Characteristic**	**Primary cohort**			**Validation cohort**		
	**Good absorption** **(*****n** **=*** **130)**	**Poor absorption** **(*****n** **=*** **66)**	**P**	**Good absorption** **(*****n** **=*** **39)**	**Poor absorption** **(*****n** **=*** **21)**	***P***
Age, median (Interquartile range), years	66.0 (54.0–75.0)	64.0 (55.5–74.0)	0.782	70.0 (60.0–75.0)	65.0 (57.0–75.0)	0.394
Gender (male): no. (%)	109 (83.8)	60 (90.9)	0.175	27 (69.2)	18 (85.7)	0.160
CSDH with TBI History: no. (%)	119 (91.5)	58 (87.9)	0.413	33 (84.6)	14 (66.7)	0.200
Hypertension: no. (%)	23 (17.7)	12 (18.2)	0.933	9 (23.1)	8 (38.1)	0.218
Diabetes mellitus: no. (%)	5 (3.8)	8 (12.1)	0.058^*^	4 (10.3)	4 (19.0)	0.577
Hyperlipidaemia: no. (%)	16 (12.3)	7 (10.6)	0.726	0 (0)	0 (0)	NA
Symptoms						
Headache: no. (%)	92 (70.8)	44 (66.7)	0.556	28 (71.8)	13 (61.9)	0.432
Weakness: no. (%)	35 (26.9)	25 (37.9)	0.116	11 (28.2)	6 (28.6)	0.976
MGS-GCS score: no. (%)			0.103			0.121
0	1	3		6	1	
1	120	55		29	14	
2	9	8		4	6	
ADL-BI score: median (Interquartile range)	95.0 (88.8–100.0)	95.0 (80.0–100.0)	0.333	100.0 (95.0–100.0)	95.0 (90.0–100.0)	0.103
Usage of atorvastatin: no. (%)	71 (54.6)	27 (40.9)	0.070^*^	39 (100.0)	21 (100.0)	NA
Hematoma volume, median (Interquartile range), ml	59.1 (36.6–86.0)	71.9 (51.1–115.5)	0.003^*^	60.1 (43.8–70.1)	68.7 (47.3–102.0)	0.032^*^
Thick of hematoma, median (Interquartile range), mm	15.0 (11.0–19.0)	15.0 (12.0–20.0)	0.542	10.0 (8.0–12.0)	15.0 (10.0–18.0)	0.011^*^
Midline shift distance, median (Interquartile range), mm	1.0 (0–5.3)	2.0 (0–7.0)	0.508	2.0 (2.0–4.5)	2.0 (4.0–6.0)	0.105
Presence of basal ganglia compress: no. (%)	27 (20.8)	24 (36.4)	0.019^*^	5 (12.8)	11 (52.4)	0.001^*^
Hematoma location (unilateral hematoma): no. (%)	99 (76.2)	40 (60.6)	0.024^*^	31 (79.5)	13 (61.9)	0.142
Presence of septate hematoma: no. (%)	19 (14.6)	4 (6.1)	0.079^*^	10 (25.6)	2 (9.5)	0.250

* *P < 0.05*.

### Feature selection and prediction model establishment

**Figure 2:**
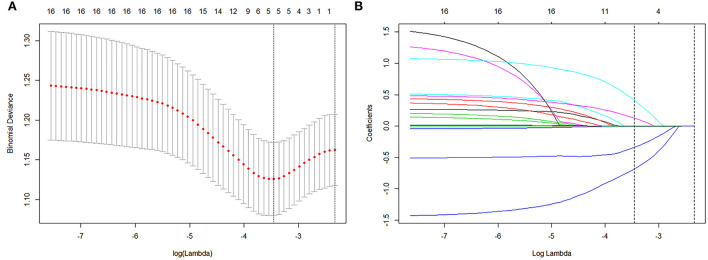
Texture feature selection using the least absolute shrinkage and selection operator (LASSO) method. **(A)** Tuning parameter (λ) selection in the LASSO model used 10-fold cross-validation *via* minimum criteria. The binomial deviance was plotted vs. log (Lambda). Dotted vertical lines were drawn at the optimal values by using the minimum criteria and the 1 standard error of the minimum criteria (the 1-SE criteria). Log (Lambda) −3.809 was chosen (1-SE criteria) according to 10-fold cross-validation. **(B)** LASSO coefficient profiles of the 15 texture features. A coefficient profile plot was produced against the log (λ) sequence. Vertical line was drawn at the value selected using a 10-fold cross-validation, where optimal λ resulted in 5 nonzero coefficients.

**Figure 3:**
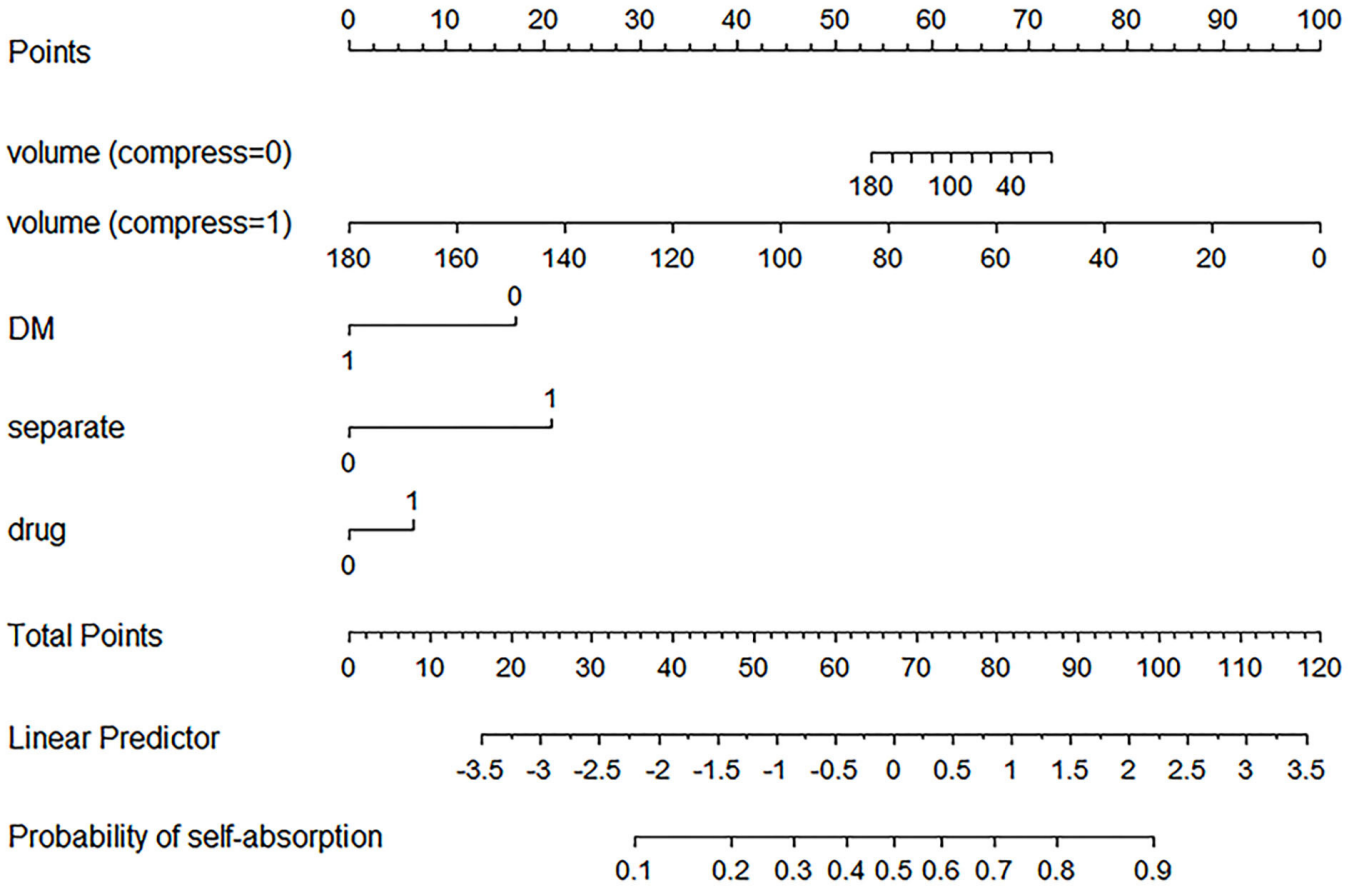
Established nomogram. The nomogram was developed in the primary cohort, with a history of diabetes mellitus (8), the use of atorvastatin (drug), hematoma volume (volume), presence of basal ganglia compress (compress), and presence of septate hematoma (separate) incorporated.

Known predictors, such as baseline volume and drug usage were associated with greater likelihood of good absorption. Of these features, 15 potential predictors of 196 patients in the primary cohort (13:1 ratio, Figure 2) were analyzed by the LASSO logistic regression model. The LASSO logistic regression analysis identified 5 independent predictors: history of diabetes mellitus (8), the use of atorvastatin (drug), hematoma volume (volume), presence of basal ganglia compress (compress), and presence of septate hematoma (separate). Incorporating the independent predictors listed above, the prediction model was developed by multivariable logistic regression analysis (Table 2) and presented as the nomogram (Figure 3).

**Table 2:** Risk factors for good self-absorption in patients with CSDH by multivariable logistic regression.

**Intercept and variable**	***β***	**Odds ratio (95% CI)**	***P***
Intercept	1.577		<0.001
History of diabetes mellitus (8)	−1.185	0.306 (0.091–1.028)	0.055
Presence of septate hematoma (separate)	1.250	3.491 (1.045–11.658)	0.042
Use of atorvastatin (drug)	0.475	1.609 (0.851–3.041)	0.143
Hematoma volume (volume)	−0.014	0.986 (0.977–0.996)	0.004
Presence of basal ganglia compress (compress)	−0.627	0.534 (0.262–1.087)	0.084

For example, a patient with CSDH has a history of diabetes mellitus, usage of atorvastatin, 60 ml of hematoma volume without basal ganglia compress and septation. Based on the nomogram, the point of volume (compress = 0) is about 65, the point of DM is 0, the point of separate is 0, and the point of drug is about 8. The total point is 73, indicating that self-absorption is approximately 60%.

### Apparent performance of the nomogram in the primary cohort

**Figure 4:**
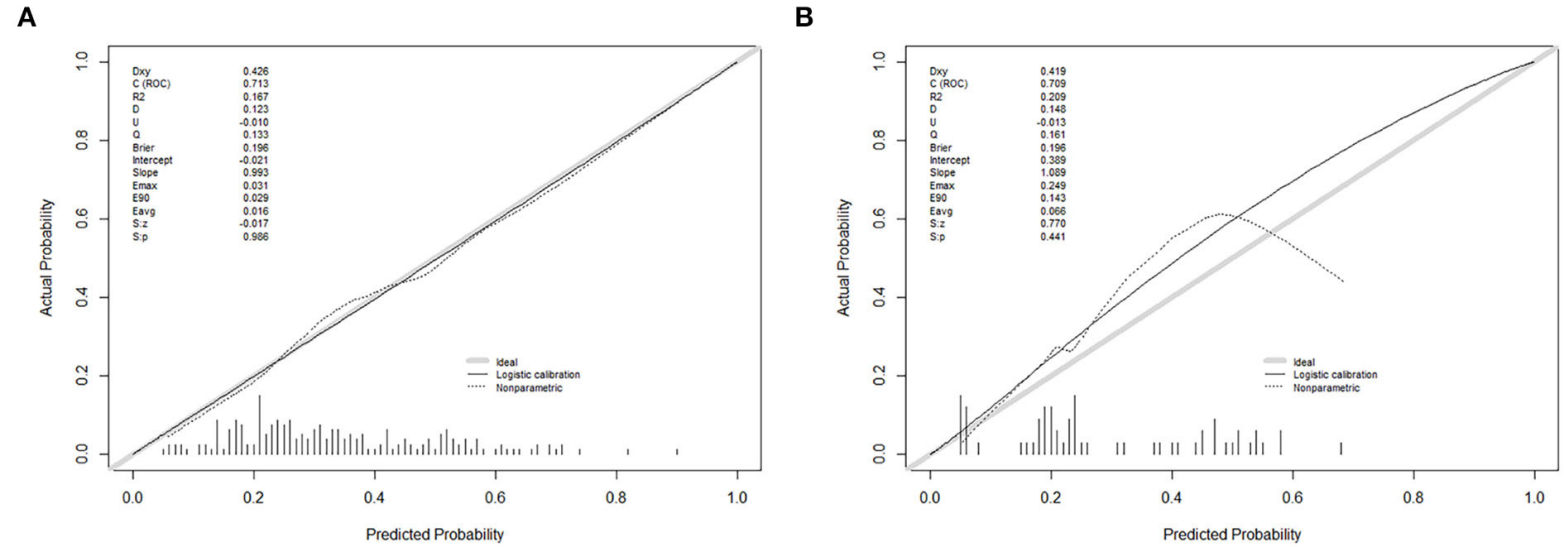
Calibration curves of the nomogram prediction in each cohort. **(A)** A calibration curve of the nomogram in the primary cohort. **(B)** A calibration curve of the nomogram in the validation cohort. Calibration curves depict the calibration of each model in terms of the agreement between the predicted probability of self-absorption and observed outcomes of self-absorption. The *y*-axis represents the actual self-absorption rate. The *x*-axis represents the predicted self-absorption probability. The diagonal gray solid line represents a perfect prediction by an ideal model. The thin black solid line represents the performance of the nomogram, of which a closer fit to the diagonal solid line represents a better prediction.

The calibration curve of the nomogram with good absorption probability showed good consistency between prediction and observation in the primary cohort (Figure 4A). The Hosmer–Lemeshow test did not present a significant difference (*p* = 0.986), which indicated that the perfect fit did not deviate. The ROC curve of the prediction nomogram indicated that the AUC was 0.713 (95% *CI*, 0.637–0.788) in the primary cohort.

### Independent validation of the nomogram

A good calibration of good absorption probability was observed in the validation cohort (Figure 4B). No significant difference could be noticed from the Hosmer–Lemeshow test (*p* = 0.441). The ROC curve for the prediction of good absorption showed that the AUC was 0.709 (95% CI, 0.574–0.844) in the validation cohort.

### Clinical use

**Figure 5:**
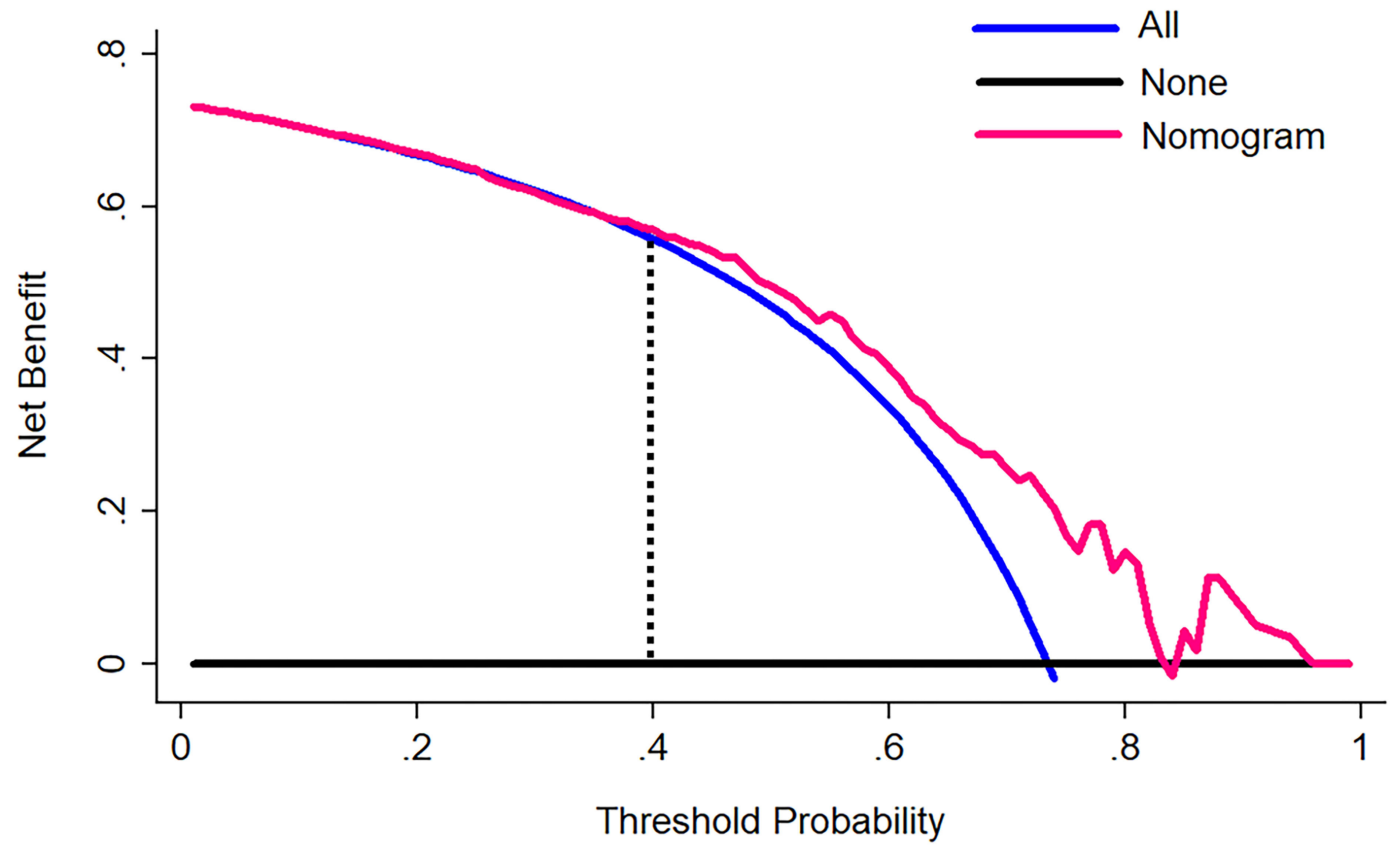
A decision curve analysis for the nomogram. The *y*-axis measures the net benefit. The pink line represents the nomogram. The blue line represents the assumption that all patients have self-absorption. The black line represents the assumption that no patients have self-absorption. The net benefit was calculated by subtracting the proportion of all patients who are false positive from the proportion who are true positive, weighting by the relative harm of forgoing treatment compared with the negative consequences of an unnecessary treatment. The decision curve showed that if the self-absorption probability of a patient >40%, using the nomogram in the current study to predict self-absorption adds more benefit than either assumption that all patients have self-absorption or assumption that no patients have self-absorption.

The decision curve for the nomogram (Figure 5) showed that if the threshold of probability was > 40% in one patient, using this model for predicting good self-absorption brings more benefits than the assumption that all patients have self-absorption or no patients have self-absorption. The prediction model can assist in guiding the choice of therapy, whether to undergo non-surgical treatment or surgery.

## Discussion

A nomogram was established and validated for the personalized prediction of hematoma self-absorption in patients with CSDH. The primary cohort was a well-designed multicenter CSDH clinical trial population, which could present the patients with CSDH in China. The nomogram incorporated five items: history of diabetes mellitus (8), the use of atorvastatin (drug), hematoma volume (volume), presence of basal ganglia compress (compress), and presence of septate hematoma (separate). We demonstrated that using an easy-to-use predictive tool (containing five items), CSDH patients with a high risk of self-absorption could undergo only conservative treatment.

For examining the predictor-outcome association, the 15 candidate features were eventually reduced to 5 potential predictors by using the LASSO method to narrow the regression coefficients. This method went beyond the method of selecting predictors based on the univariate intensity associated with the outcome (10). The nomogram that combined multiple individual features demonstrated adequate discrimination in the primary cohort (AUC, 0.713), which was very similar in the validation cohort (AUC, 0.709). Given that the good absorption was comparable in the two cohorts, the similar discrimination implied that the nomogram was robust for prediction and could be applied directly in the validation cohort.

Although the pathogenesis of CSDH was studied by a succession of studies, which aspects included pontine vein avulsion hemorrhage, increased osmolality, hematoma capsule bleeding, and local hyperfibrinolysis, the pathogenesis and absorption mechanism of CSDH were not well understood (14, 15). The amount of inflammatory cytokines and vascular endothelial growth factor (VEGF) secreted into hematoma, leading to the proliferation of the immature blood vessels on the capsule, the damage of vascular endothelial cells, and the opening of gap junctions. The increased permeability causes the continuous leakage of circulating substances, resulting in the gradual hematoma growth (15). Meanwhile, the key influence factor in the formation process of CSDH may be the missing of anti-inflammatory and pro-repair components, such as regulatory T (Treg) cells and endothelial progenitor cells (EPCs), which lead to the recurrence of “immature angiogenesis—endothelial cell damage—vascular leakage” on hematoma walls (14, 16–19). In the formation and development of CSDH, immunomodulatory abnormalities and decreased vascular repair maturation have been confirmed playing a critical role by related studies (20–22).

It is well-known that diabetes mellitus is a prominent risk factor for coronary heart disease and stroke (23). Our results showed that the presence of diabetes mellitus would reduce the probability of hematoma self-absorption. We considered that diabetes mellitus might weaken the neovascularization on the hematoma wall, leading to a gradual increase in hematoma growth due to continuous leakage greater than absorption. Similarly, Pang et al. (24) reported that the diabetes mellitus was related to an increased likelihood of recurrence.

Statins, also known as selective HMG-CoA reductase inhibitors, have previously been applied to treat hyperlipidemia. They have been widely used in the treatment of cardiovascular disease. Currently, some studies had proved that atorvastatin could promote the absorption of CSDH and prevent the recurrence of CSDH (25–27). Our previous RCT study, which was completed by 25 Chinese neurosurgery centers indicated that the hematoma volume reduction in the atorvastatin group was 12.55 ml more compared with the control group after low-dose (20 mg/days) and long-term (8 weeks) treatment. Neurologic symptoms in most patients improved significantly after drug therapy. The transfer to surgery rate of the atorvastatin treatment group was also found to be significantly reduced (6).

The hematoma capsule is very important in which both bleeding and reabsorption occur. El-Kadi et al. (28) demonstrated that the larger the CSDH was, the lower the capsule/volume ratio would appear. Therefore, hematoma volume is the negative factor for self-absorption. Basal ganglia compress is accompanied by hematoma volume enlargement, so their effects are consistent. We have considered the interaction between hematoma volume and basal ganglia compress in the nomogram. Conversely, a CSDH with septate hematoma has a greater surface/volume ratio. It followed that the existence of septate hematoma was a positive factor for self-absorption, which was consistent with previously reported results (29).

The most important part of using the nomogram is the requirement to explain operations clearly based on individual need. The difficulty we face lies in being unable to capture the clinical consequences of a particular level of discrimination or degree of miscalculation (30, 31). Therefore, we assessed whether the nomogram-assisted decision-making would reduce unnecessary surgery and further enhance patient prognosis to confirm clinical utility. At this moment, multi-institutional prospective validation of the nomogram is very necessary. But it is difficult to implement due to the heterogeneity of clinical data collection and CT image acquisition in different centers. As a result, this study used decision curve analysis. This new approach pays attention to clinical consequences according to threshold probabilities, from which a net benefit can be expressed. The net benefit is calculated by subtracting the proportion of false positives from the proportion of false positives and weighted based on the relative harm of false positives and false negative results (13). In the case of a patient's threshold probability of >40%, the decision curve suggested that the nomogram in the current study could be used to predict hematoma absorption. This approach added more support than the hypothesis that all patients had self-absorption or no patients had self-absorption.

Some limitations should be considered in this paper. First, to maximize the enrollment of clinical trials (Efficacy of Atorvastatin on Chronic Subdural Hematoma), which as the primary cohort, we selected CSDH patients with relatively small baseline hematoma volume. Hematoma volume is known as the main influenced factor of hematoma absorption (28), which was similar in our study. Hence, our results might have bias because the proportion of good absorption was approximately 3 times more than poor absorption in the primary cohort. Second, patients in the primary cohort was absent from anticoagulant drugs, which is a known predictor of hematoma growth (32). This prediction model was only applied for CSDH patients without the usage of anticoagulant drugs, while we suggested that operation was the first choice for CSDH patients with the usage of anticoagulant drugs after correcting the coagulation function. Third, this prediction model was developed based on Chinese patients, so whether it could be used to other races may need further studies.

In conclusion, this study proposed a nomogram that combined clinical features and CT image features, which could be conveniently used to predict the probability of CSDH self-absorption and reduce unnecessary manipulation.

## Oriental neurosurgical evidence-based study team (ONET) in china

Tianjin Medical University General Hospital: Shuyuan Yue. First Affiliated Hospital of Harbin Medical University: Shiguang Zhao. Peking Union Medical College Hospital: Renzhi Wang and Junji Wei. Southwest Hospital: Hua Feng and Rong Hu. Second Affiliated Hospital, Zhejiang University School of Medicine: Jianmin Zhang. Qilu Hospital of Shandong University: Xingang Li. Huashan Hospital Fudan University: Ying Mao and Jin Hu. Xiangya Hospital of Central South University: Xianrui Yuan. Xijing Hospital of the fourth Military Medical University: Zhou Fei. Beijing TianTan Hospital, the Capital Medical University: Yuanli Zhao. General Hospital of Chinese People's Liberation Army: Xingang Yu. Prince of Wales Hospital, Chinese University of Hong Kong: Wai Sang Poon. Linyi People's Hospital: Xinde Zhu. Jiangsu Provincial Hospital, Nanjing Medical University First Affiliated Hospital: Ning Liu. First Affiliated Hospital of Fujian Medical University: Dezhi Kang. General Hospital of Ningxia Medical University: Tao Sun. Second Affiliated Hospital of Hebei Medical University: Baohua Jiao. First Affiliated Hospital of Zhengzhou University: Xianzhi Liu. Affiliated Hospital of Xuzhou Medical College: Rutong Yu. Central Hospital of Erdos: Junyi Zhang. Xi'an Tangdu Hospital of the fourth Military Medical University: Guodong Gao. First Affiliated Hospital of Shanxi Medical University: Jiehe Hao. Provincial People's Hospital of Inner Mongolia: Ning Su. Cangzhou Central Hospital: Gangfeng Yin. Second Affiliated Hospital of Nanchang University: Xingen Zhu. Shanghai Changzheng Hospital: Yicheng Lu. 117th Hospital of Chinese People's Liberation Army: Jianrong Li.

## Data availability statement

The raw data supporting the conclusions of this article will be made available by the authors, without undue reservation.

## Ethics statement

The studies involving human participants were reviewed and approved by Tianjin Medical University General Hospital. The patients/participants provided their written informed consent to participate in this study.

## Author contributions

JS, RJ, and JZ had full access to all of the data in the study and take responsibility for the integrity of the data and the accuracy of the data analysis. RJ and JZ: concept and design. YT, DW, and XZ: acquisition, analysis, or interpretation of data. YT: drafting of the manuscript. HW, YW, and SA: critical revision of the manuscript for important intellectual content. CG and JH: statistical analysis. YT, DW, RJ, and JZ: obtained funding. All authors contributed to the article and approved the submitted version.

## Funding

The National Natural Science Foundation of China, 81971173 and 81501057, YT received the funding. The National Natural Science Foundation of China, 82171359 and 81671380 and Beijing-Tianjin-Hebei Cooperation Project, 19JCZDJC64600(Z), DW received the funding. The State key Program of the National Natural Science Foundation of China, 81930031, JZ received the funding. Tianjin Research Program of Application Foundation and Advanced Technology, 19YFZCSY00650, RJ received the funding. ATOCH trial was supported by the Chinese Society of Neurosurgery, Chinese Medical Association and Zhao Yi-Cheng Medical Science Foundation (http://www.zycmsf.com/en/index.asp).

## Conflict of interest

The authors declare that the research was conducted in the absence of any commercial or financial relationships that could be construed as a potential conflict of interest.

## Publisher's note

All claims expressed in this article are solely those of the authors and do not necessarily represent those of their affiliated organizations, or those of the publisher, the editors and the reviewers. Any product that may be evaluated in this article, or claim that may be made by its manufacturer, is not guaranteed or endorsed by the publisher.
